# Dichloridobis(4-chloro­benzyl-κ*C*)(1,10-phenanthroline-κ^2^ *N*,*N*′)tin(IV)

**DOI:** 10.1107/S1600536809019795

**Published:** 2009-06-06

**Authors:** Chui Lian Tan, Kong Mun Lo, Seik Weng Ng

**Affiliations:** a Department of Chemistry, University of Malaya, 50603 Kuala Lumpur, Malaysia

## Abstract

In the crystal structure of the title compound, [Sn(C_7_H_6_Cl)_2_Cl_2_(C_12_H_8_N_2_)], the Sn^IV^ atom is chelated by the *N*-heterocycle and the metal atom exists in a *trans*-C_2_SnCl_2_N_2_ distorted octa­hedral coordination environment.

## Related literature

Several diorganotin dichloride adducts of 2,2′-bipyridine have been reported. For the diethyl­tin dichloride, dibutyl­tin dichoride and dibenzyl­tin dichloride adducts; see Chadha *et al.* (1980 ▶); Gill *et al.*(1999 ▶); Tiekink *et al.* (2000 ▶). For the structure of di(4-chloro­benzyl­tin) dichloride, see: Kuang & Feng (2000 ▶). For the direct synthesis of di(chloro­benz­yl)tin dichlorides, see: Sisido *et al.* (1961 ▶).
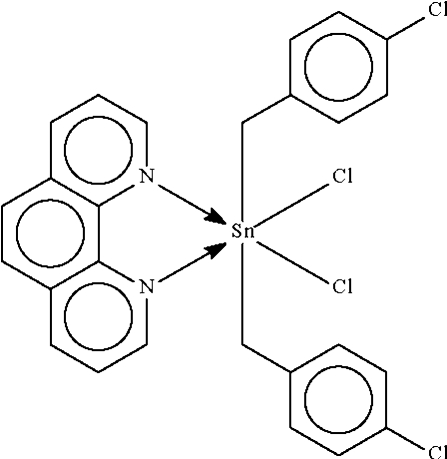


## Experimental

### 

#### Crystal data

[Sn(C_7_H_6_Cl)_2_Cl_2_(C_12_H_8_N_2_)]
*M* *_r_* = 620.93
Monoclinic, 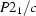

*a* = 8.9252 (1) Å
*b* = 17.9987 (3) Å
*c* = 15.6862 (3) Å
β = 98.686 (1)°
*V* = 2490.96 (7) Å^3^
*Z* = 4
Mo *K*α radiation
μ = 1.47 mm^−1^
*T* = 119 K
0.40 × 0.10 × 0.10 mm

#### Data collection

Bruker SMART APEX diffractometer
Absorption correction: multi-scan (*SADABS*; Sheldrick, 1996 ▶) *T*_min_ = 0.590, *T*_max_ = 0.867
16905 measured reflections
5681 independent reflections
4826 reflections with *I* > 2σ(*I*)
*R*_int_ = 0.021

#### Refinement

*R*[*F* ^2^ > 2σ(*F* ^2^)] = 0.023
*wR*(*F* ^2^) = 0.062
*S* = 1.05
5681 reflections
298 parameters
H-atom parameters constrained
Δρ_max_ = 0.53 e Å^−3^
Δρ_min_ = −0.44 e Å^−3^

### 

Data collection: *APEX2* (Bruker, 2007 ▶); cell refinement: *SAINT* (Bruker, 2007 ▶); data reduction: *SAINT*; program(s) used to solve structure: *SHELXS97* (Sheldrick, 2008 ▶); program(s) used to refine structure: *SHELXL97* (Sheldrick, 2008 ▶); molecular graphics: *X-SEED* (Barbour, 2001 ▶); software used to prepare material for publication: *publCIF* (Westrip, 2009 ▶).

## Supplementary Material

Crystal structure: contains datablocks global, I. DOI: 10.1107/S1600536809019795/xu2530sup1.cif

Structure factors: contains datablocks I. DOI: 10.1107/S1600536809019795/xu2530Isup2.hkl

Additional supplementary materials:  crystallographic information; 3D view; checkCIF report

## Figures and Tables

**Table:** 

Sn1—C1	2.160 (2)
Sn1—C8	2.162 (2)
Sn1—N1	2.3712 (18)
Sn1—N2	2.3515 (18)
Sn1—Cl3	2.5287 (6)
Sn1—Cl4	2.4973 (6)

**Table:** 

C1—Sn1—C8	172.84 (8)

**Fig. 1.**
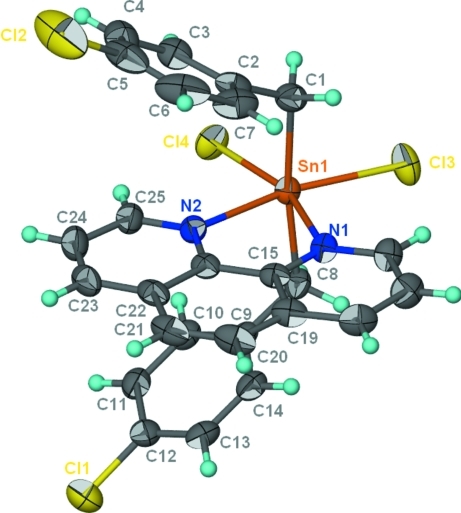
The molecular structure of the title compound with 70% displacement probability ellipsoids. Hydrogen atoms are drawn as spheres of arbitrary radius.

## supplementary crystallographic information

### Experimental

Di(*p*-chlorobenzyl)tin dichloride was synthesized by the reaction of *p*-chlorobenzyl chloride and metallic tin (Sisido *et al.*, 1961). The reactant (0.5 g, 1.1 mmol) and 1,10-phenanthroline (0.2 g, 1.1 mmol) were heated in chloroform (50 ml) for 1 hour. Faint-yellow crystals separated from the cool solution after a day.

### Refinement

Hydrogen atoms were placed at calculated positions (C–H 0.95–0.99 Å) and were treated as riding on their parent atoms, with *U*(H) set to 1.2 times *U*_eq_(C).

### Crystal data

**Table:**

[Sn(C_7_H_6_Cl)_2_Cl_2_(C_12_H_8_N_2_)]	*F*(000) = 1232
*M**_r_* = 620.93	*D*_x_ = 1.656 Mg m^−^^3^
Monoclinic, *P*2_1_/*c*	Mo *K*α radiation, λ = 0.71073 Å
Hall symbol: -P 2ybc	Cell parameters from 8381 reflections
*a* = 8.9252 (1) Å	θ = 2.3–28.2°
*b* = 17.9987 (3) Å	µ = 1.47 mm^−^^1^
*c* = 15.6862 (3) Å	*T* = 119 K
β = 98.686 (1)°	Prism, yellow
*V* = 2490.96 (7) Å^3^	0.40 × 0.10 × 0.10 mm
*Z* = 4	

### Data collection

**Table:**

Bruker SMART APEX diffractometer	5681 independent reflections
Radiation source: fine-focus sealed tube	4826 reflections with *I* > 2σ(*I*)
graphite	*R*_int_ = 0.021
ω scans	θ_max_ = 27.5°, θ_min_ = 1.7°
Absorption correction: multi-scan (*SADABS*; Sheldrick, 1996)	*h* = −11→11
*T*_min_ = 0.590, *T*_max_ = 0.867	*k* = −22→23
16905 measured reflections	*l* = −20→20

### Refinement

**Table:**

Refinement on *F*^2^	Primary atom site location: structure-invariant direct methods
Least-squares matrix: full	Secondary atom site location: difference Fourier map
*R*[*F*^2^ > 2σ(*F*^2^)] = 0.023	Hydrogen site location: inferred from neighbouring sites
*wR*(*F*^2^) = 0.062	H-atom parameters constrained
*S* = 1.05	*w* = 1/[σ^2^(*F*_o_^2^) + (0.0286*P*)^2^ + 1.4291*P*] where *P* = (*F*_o_^2^ + 2*F*_c_^2^)/3
5681 reflections	(Δ/σ)_max_ = 0.001
298 parameters	Δρ_max_ = 0.53 e Å^−^^3^
0 restraints	Δρ_min_ = −0.44 e Å^−^^3^

### Fractional atomic coordinates and isotropic or equivalent isotropic displacement parameters (Å^2^)

**Table:**

	*x*	*y*	*z*	*U*_iso_*/*U*_eq_	
Sn1	0.668417 (16)	0.554696 (8)	0.795225 (9)	0.02121 (5)	
Cl1	0.84702 (8)	0.57955 (4)	0.35794 (4)	0.04106 (15)	
Cl2	1.35725 (11)	0.75339 (6)	0.97376 (6)	0.0765 (3)	
Cl3	0.42667 (7)	0.52254 (3)	0.85279 (4)	0.03635 (14)	
Cl4	0.82575 (7)	0.43941 (3)	0.81282 (4)	0.03295 (13)	
N1	0.5902 (2)	0.67771 (10)	0.75699 (12)	0.0231 (4)	
N2	0.8592 (2)	0.61534 (10)	0.73338 (12)	0.0232 (4)	
C1	0.7565 (3)	0.59581 (14)	0.92228 (15)	0.0302 (5)	
H1A	0.6811	0.6304	0.9405	0.036*	
H1B	0.7663	0.5533	0.9628	0.036*	
C2	0.9049 (3)	0.63463 (13)	0.93108 (14)	0.0291 (5)	
C3	1.0409 (3)	0.59536 (13)	0.94752 (15)	0.0313 (5)	
H3A	1.0382	0.5427	0.9503	0.038*	
C4	1.1800 (3)	0.63119 (16)	0.95997 (17)	0.0399 (6)	
H4A	1.2716	0.6037	0.9725	0.048*	
C5	1.1823 (4)	0.70741 (17)	0.95385 (17)	0.0444 (7)	
C6	1.0518 (4)	0.74826 (15)	0.93470 (18)	0.0499 (8)	
H6A	1.0560	0.8007	0.9292	0.060*	
C7	0.9140 (3)	0.71175 (14)	0.92356 (16)	0.0399 (6)	
H7A	0.8232	0.7398	0.9105	0.048*	
C8	0.5528 (2)	0.51792 (13)	0.67136 (15)	0.0259 (5)	
H8A	0.5366	0.4636	0.6746	0.031*	
H8B	0.4516	0.5416	0.6615	0.031*	
C9	0.6284 (2)	0.53300 (12)	0.59490 (14)	0.0236 (4)	
C10	0.7343 (3)	0.48429 (13)	0.56968 (15)	0.0281 (5)	
H10A	0.7604	0.4406	0.6025	0.034*	
C11	0.8025 (3)	0.49820 (13)	0.49766 (15)	0.0294 (5)	
H11A	0.8738	0.4641	0.4808	0.035*	
C12	0.7655 (3)	0.56232 (13)	0.45046 (15)	0.0290 (5)	
C13	0.6620 (3)	0.61251 (13)	0.47410 (15)	0.0301 (5)	
H13A	0.6378	0.6566	0.4416	0.036*	
C14	0.5942 (3)	0.59752 (13)	0.54598 (15)	0.0281 (5)	
H14A	0.5228	0.6318	0.5625	0.034*	
C15	0.6868 (2)	0.71888 (11)	0.71740 (14)	0.0221 (4)	
C16	0.4601 (3)	0.70774 (13)	0.77128 (15)	0.0277 (5)	
H16A	0.3933	0.6790	0.7998	0.033*	
C17	0.4182 (3)	0.78056 (13)	0.74554 (17)	0.0334 (5)	
H17A	0.3249	0.8007	0.7569	0.040*	
C18	0.5130 (3)	0.82184 (13)	0.70422 (16)	0.0325 (5)	
H18A	0.4846	0.8707	0.6852	0.039*	
C19	0.6528 (3)	0.79239 (12)	0.68953 (15)	0.0281 (5)	
C20	0.7614 (3)	0.83310 (13)	0.64909 (15)	0.0346 (6)	
H20A	0.7388	0.8825	0.6299	0.041*	
C21	0.8942 (3)	0.80256 (14)	0.63798 (15)	0.0337 (6)	
H21A	0.9643	0.8308	0.6114	0.040*	
C22	0.9325 (3)	0.72786 (13)	0.66552 (14)	0.0282 (5)	
C23	1.0714 (3)	0.69379 (16)	0.65643 (16)	0.0348 (6)	
H23A	1.1443	0.7199	0.6297	0.042*	
C24	1.1010 (3)	0.62301 (15)	0.68614 (16)	0.0356 (6)	
H24A	1.1948	0.5998	0.6810	0.043*	
C25	0.9913 (3)	0.58531 (14)	0.72420 (15)	0.0288 (5)	
H25A	1.0124	0.5361	0.7443	0.035*	
C26	0.8288 (2)	0.68613 (12)	0.70487 (14)	0.0231 (4)	

### Atomic displacement parameters (Å^2^)

**Table:**

	*U*^11^	*U*^22^	*U*^33^	*U*^12^	*U*^13^	*U*^23^
Sn1	0.02126 (8)	0.01781 (8)	0.02478 (8)	0.00048 (6)	0.00414 (6)	−0.00034 (6)
Cl1	0.0462 (4)	0.0487 (4)	0.0299 (3)	−0.0001 (3)	0.0111 (3)	0.0010 (3)
Cl2	0.0833 (6)	0.0975 (7)	0.0515 (5)	−0.0638 (6)	0.0192 (5)	−0.0244 (5)
Cl3	0.0361 (3)	0.0285 (3)	0.0493 (4)	−0.0040 (2)	0.0220 (3)	−0.0024 (3)
Cl4	0.0322 (3)	0.0230 (3)	0.0424 (3)	0.0073 (2)	0.0017 (3)	0.0020 (2)
N1	0.0221 (9)	0.0201 (9)	0.0271 (10)	−0.0018 (7)	0.0033 (8)	−0.0027 (7)
N2	0.0204 (9)	0.0261 (10)	0.0226 (9)	−0.0026 (7)	0.0019 (7)	−0.0039 (7)
C1	0.0345 (13)	0.0315 (12)	0.0244 (11)	0.0055 (10)	0.0041 (10)	−0.0019 (10)
C2	0.0420 (14)	0.0268 (12)	0.0179 (11)	−0.0005 (10)	0.0033 (10)	−0.0027 (9)
C3	0.0388 (13)	0.0265 (12)	0.0287 (12)	−0.0032 (10)	0.0053 (10)	−0.0051 (10)
C4	0.0391 (14)	0.0490 (16)	0.0327 (14)	−0.0081 (12)	0.0083 (12)	−0.0129 (12)
C5	0.0597 (19)	0.0497 (17)	0.0246 (13)	−0.0293 (15)	0.0090 (13)	−0.0113 (12)
C6	0.087 (2)	0.0287 (14)	0.0304 (14)	−0.0172 (15)	−0.0030 (15)	−0.0025 (11)
C7	0.0597 (18)	0.0270 (13)	0.0296 (13)	0.0023 (12)	−0.0038 (12)	−0.0051 (10)
C8	0.0195 (10)	0.0280 (12)	0.0296 (12)	−0.0026 (9)	0.0018 (9)	−0.0041 (9)
C9	0.0186 (10)	0.0264 (11)	0.0237 (11)	−0.0024 (8)	−0.0037 (9)	−0.0047 (9)
C10	0.0253 (11)	0.0274 (12)	0.0298 (12)	0.0006 (9)	−0.0015 (10)	−0.0026 (9)
C11	0.0244 (11)	0.0325 (12)	0.0304 (12)	0.0031 (9)	0.0015 (10)	−0.0047 (10)
C12	0.0266 (11)	0.0369 (13)	0.0227 (11)	−0.0023 (10)	0.0004 (9)	−0.0024 (10)
C13	0.0303 (12)	0.0295 (12)	0.0275 (12)	0.0033 (10)	−0.0052 (10)	0.0006 (10)
C14	0.0263 (11)	0.0272 (12)	0.0290 (12)	0.0049 (9)	−0.0019 (9)	−0.0043 (9)
C15	0.0269 (11)	0.0198 (10)	0.0186 (10)	−0.0044 (8)	0.0004 (8)	−0.0030 (8)
C16	0.0256 (11)	0.0242 (11)	0.0336 (13)	0.0000 (9)	0.0053 (10)	−0.0026 (9)
C17	0.0349 (13)	0.0254 (12)	0.0396 (14)	0.0061 (10)	0.0042 (11)	−0.0042 (10)
C18	0.0448 (14)	0.0188 (11)	0.0326 (13)	0.0041 (10)	0.0016 (11)	0.0003 (9)
C19	0.0387 (13)	0.0200 (11)	0.0247 (11)	−0.0039 (9)	0.0018 (10)	−0.0028 (9)
C20	0.0540 (16)	0.0229 (12)	0.0271 (12)	−0.0084 (11)	0.0072 (11)	0.0010 (9)
C21	0.0447 (15)	0.0321 (13)	0.0248 (12)	−0.0179 (11)	0.0072 (11)	−0.0028 (10)
C22	0.0311 (12)	0.0347 (13)	0.0186 (11)	−0.0118 (10)	0.0030 (9)	−0.0041 (9)
C23	0.0262 (12)	0.0531 (16)	0.0260 (12)	−0.0147 (11)	0.0066 (10)	−0.0045 (11)
C24	0.0233 (11)	0.0501 (16)	0.0341 (13)	−0.0029 (11)	0.0070 (10)	−0.0070 (12)
C25	0.0233 (11)	0.0351 (12)	0.0280 (12)	0.0001 (10)	0.0036 (9)	−0.0055 (10)
C26	0.0250 (11)	0.0245 (11)	0.0190 (10)	−0.0059 (9)	0.0011 (8)	−0.0045 (8)

### Geometric parameters (Å, °)

**Table:**

Sn1—C1	2.160 (2)	C9—C14	1.400 (3)
Sn1—C8	2.162 (2)	C10—C11	1.385 (3)
Sn1—N1	2.3712 (18)	C10—H10A	0.9500
Sn1—N2	2.3515 (18)	C11—C12	1.384 (3)
Sn1—Cl3	2.5287 (6)	C11—H11A	0.9500
Sn1—Cl4	2.4973 (6)	C12—C13	1.382 (3)
Cl1—C12	1.747 (2)	C13—C14	1.385 (3)
Cl2—C5	1.753 (3)	C13—H13A	0.9500
N1—C16	1.330 (3)	C14—H14A	0.9500
N1—C15	1.356 (3)	C15—C19	1.412 (3)
N2—C25	1.325 (3)	C15—C26	1.438 (3)
N2—C26	1.364 (3)	C16—C17	1.405 (3)
C1—C2	1.486 (3)	C16—H16A	0.9500
C1—H1A	0.9900	C17—C18	1.361 (4)
C1—H1B	0.9900	C17—H17A	0.9500
C2—C3	1.394 (3)	C18—C19	1.406 (3)
C2—C7	1.397 (3)	C18—H18A	0.9500
C3—C4	1.386 (4)	C19—C20	1.437 (3)
C3—H3A	0.9500	C20—C21	1.342 (4)
C4—C5	1.376 (4)	C20—H20A	0.9500
C4—H4A	0.9500	C21—C22	1.437 (4)
C5—C6	1.372 (4)	C21—H21A	0.9500
C6—C7	1.382 (4)	C22—C26	1.405 (3)
C6—H6A	0.9500	C22—C23	1.410 (3)
C7—H7A	0.9500	C23—C24	1.368 (4)
C8—C9	1.487 (3)	C23—H23A	0.9500
C8—H8A	0.9900	C24—C25	1.397 (3)
C8—H8B	0.9900	C24—H24A	0.9500
C9—C10	1.390 (3)	C25—H25A	0.9500
			
C1—Sn1—C8	172.84 (8)	C14—C9—C8	120.4 (2)
C8—Sn1—N2	92.78 (7)	C11—C10—C9	121.3 (2)
C1—Sn1—N2	92.08 (8)	C11—C10—H10A	119.3
C8—Sn1—N1	88.64 (8)	C9—C10—H10A	119.3
C1—Sn1—N1	88.03 (8)	C10—C11—C12	119.3 (2)
N2—Sn1—N1	70.49 (6)	C10—C11—H11A	120.4
C8—Sn1—Cl4	91.67 (6)	C12—C11—H11A	120.4
C1—Sn1—Cl4	93.58 (7)	C13—C12—C11	121.1 (2)
N2—Sn1—Cl4	90.07 (5)	C13—C12—Cl1	119.07 (19)
N1—Sn1—Cl4	160.55 (5)	C11—C12—Cl1	119.81 (18)
C8—Sn1—Cl3	86.19 (6)	C14—C13—C12	118.9 (2)
C1—Sn1—Cl3	87.72 (7)	C14—C13—H13A	120.6
N2—Sn1—Cl3	164.47 (5)	C12—C13—H13A	120.6
N1—Sn1—Cl3	93.99 (5)	C13—C14—C9	121.5 (2)
Cl4—Sn1—Cl3	105.44 (2)	C13—C14—H14A	119.3
C16—N1—C15	119.11 (19)	C9—C14—H14A	119.3
C16—N1—Sn1	124.61 (15)	N1—C15—C19	122.1 (2)
C15—N1—Sn1	116.28 (14)	N1—C15—C26	118.23 (19)
C25—N2—C26	118.79 (19)	C19—C15—C26	119.6 (2)
C25—N2—Sn1	124.56 (16)	N1—C16—C17	122.1 (2)
C26—N2—Sn1	116.65 (14)	N1—C16—H16A	118.9
C2—C1—Sn1	116.24 (15)	C17—C16—H16A	118.9
C2—C1—H1A	108.2	C18—C17—C16	119.3 (2)
Sn1—C1—H1A	108.2	C18—C17—H17A	120.4
C2—C1—H1B	108.2	C16—C17—H17A	120.4
Sn1—C1—H1B	108.2	C17—C18—C19	120.2 (2)
H1A—C1—H1B	107.4	C17—C18—H18A	119.9
C3—C2—C7	117.4 (2)	C19—C18—H18A	119.9
C3—C2—C1	121.2 (2)	C18—C19—C15	117.2 (2)
C7—C2—C1	121.4 (2)	C18—C19—C20	123.7 (2)
C4—C3—C2	121.7 (2)	C15—C19—C20	119.1 (2)
C4—C3—H3A	119.1	C21—C20—C19	121.1 (2)
C2—C3—H3A	119.1	C21—C20—H20A	119.4
C5—C4—C3	118.5 (3)	C19—C20—H20A	119.4
C5—C4—H4A	120.8	C20—C21—C22	121.2 (2)
C3—C4—H4A	120.8	C20—C21—H21A	119.4
C6—C5—C4	121.9 (3)	C22—C21—H21A	119.4
C6—C5—Cl2	119.3 (2)	C26—C22—C23	117.4 (2)
C4—C5—Cl2	118.7 (3)	C26—C22—C21	119.3 (2)
C5—C6—C7	118.9 (3)	C23—C22—C21	123.3 (2)
C5—C6—H6A	120.6	C24—C23—C22	119.9 (2)
C7—C6—H6A	120.6	C24—C23—H23A	120.1
C6—C7—C2	121.6 (3)	C22—C23—H23A	120.1
C6—C7—H7A	119.2	C23—C24—C25	119.1 (2)
C2—C7—H7A	119.2	C23—C24—H24A	120.5
C9—C8—Sn1	117.12 (14)	C25—C24—H24A	120.5
C9—C8—H8A	108.0	N2—C25—C24	122.7 (2)
Sn1—C8—H8A	108.0	N2—C25—H25A	118.6
C9—C8—H8B	108.0	C24—C25—H25A	118.6
Sn1—C8—H8B	108.0	N2—C26—C22	122.1 (2)
H8A—C8—H8B	107.3	N2—C26—C15	118.27 (19)
C10—C9—C14	118.0 (2)	C22—C26—C15	119.6 (2)
C10—C9—C8	121.7 (2)		
			
C8—Sn1—N1—C16	88.53 (19)	C10—C11—C12—C13	0.0 (4)
C1—Sn1—N1—C16	−85.14 (19)	C10—C11—C12—Cl1	−178.41 (18)
N2—Sn1—N1—C16	−178.0 (2)	C11—C12—C13—C14	−0.4 (3)
Cl4—Sn1—N1—C16	179.68 (13)	Cl1—C12—C13—C14	178.03 (18)
Cl3—Sn1—N1—C16	2.44 (18)	C12—C13—C14—C9	0.1 (3)
C8—Sn1—N1—C15	−91.05 (16)	C10—C9—C14—C13	0.6 (3)
C1—Sn1—N1—C15	95.29 (16)	C8—C9—C14—C13	−179.4 (2)
N2—Sn1—N1—C15	2.38 (14)	C16—N1—C15—C19	−1.0 (3)
Cl4—Sn1—N1—C15	0.1 (3)	Sn1—N1—C15—C19	178.60 (16)
Cl3—Sn1—N1—C15	−177.13 (15)	C16—N1—C15—C26	178.2 (2)
C8—Sn1—N2—C25	−94.31 (18)	Sn1—N1—C15—C26	−2.2 (2)
C1—Sn1—N2—C25	90.94 (18)	C15—N1—C16—C17	0.9 (3)
N1—Sn1—N2—C25	178.12 (19)	Sn1—N1—C16—C17	−178.64 (17)
Cl4—Sn1—N2—C25	−2.64 (17)	N1—C16—C17—C18	0.4 (4)
Cl3—Sn1—N2—C25	179.92 (14)	C16—C17—C18—C19	−1.6 (4)
C8—Sn1—N2—C26	85.20 (16)	C17—C18—C19—C15	1.5 (3)
C1—Sn1—N2—C26	−89.54 (16)	C17—C18—C19—C20	−177.8 (2)
N1—Sn1—N2—C26	−2.37 (14)	N1—C15—C19—C18	−0.2 (3)
Cl4—Sn1—N2—C26	176.87 (15)	C26—C15—C19—C18	−179.4 (2)
Cl3—Sn1—N2—C26	−0.6 (3)	N1—C15—C19—C20	179.1 (2)
N2—Sn1—C1—C2	−8.28 (18)	C26—C15—C19—C20	0.0 (3)
N1—Sn1—C1—C2	−78.67 (18)	C18—C19—C20—C21	178.9 (2)
Cl4—Sn1—C1—C2	81.92 (17)	C15—C19—C20—C21	−0.4 (4)
Cl3—Sn1—C1—C2	−172.74 (18)	C19—C20—C21—C22	0.4 (4)
Sn1—C1—C2—C3	−86.5 (2)	C20—C21—C22—C26	0.0 (3)
Sn1—C1—C2—C7	93.7 (2)	C20—C21—C22—C23	−179.0 (2)
C7—C2—C3—C4	2.9 (4)	C26—C22—C23—C24	−0.5 (3)
C1—C2—C3—C4	−176.9 (2)	C21—C22—C23—C24	178.4 (2)
C2—C3—C4—C5	−1.5 (4)	C22—C23—C24—C25	0.8 (4)
C3—C4—C5—C6	−0.9 (4)	C26—N2—C25—C24	−0.3 (3)
C3—C4—C5—Cl2	177.49 (19)	Sn1—N2—C25—C24	179.23 (17)
C4—C5—C6—C7	1.8 (4)	C23—C24—C25—N2	−0.4 (4)
Cl2—C5—C6—C7	−176.7 (2)	C25—N2—C26—C22	0.5 (3)
C5—C6—C7—C2	−0.2 (4)	Sn1—N2—C26—C22	−179.00 (16)
C3—C2—C7—C6	−2.1 (4)	C25—N2—C26—C15	−178.26 (19)
C1—C2—C7—C6	177.7 (2)	Sn1—N2—C26—C15	2.2 (2)
N2—Sn1—C8—C9	5.87 (17)	C23—C22—C26—N2	−0.1 (3)
N1—Sn1—C8—C9	76.26 (17)	C21—C22—C26—N2	−179.2 (2)
Cl4—Sn1—C8—C9	−84.29 (17)	C23—C22—C26—C15	178.7 (2)
Cl3—Sn1—C8—C9	170.35 (17)	C21—C22—C26—C15	−0.4 (3)
Sn1—C8—C9—C10	86.7 (2)	N1—C15—C26—N2	0.0 (3)
Sn1—C8—C9—C14	−93.2 (2)	C19—C15—C26—N2	179.24 (19)
C14—C9—C10—C11	−1.0 (3)	N1—C15—C26—C22	−178.8 (2)
C8—C9—C10—C11	179.0 (2)	C19—C15—C26—C22	0.4 (3)
C9—C10—C11—C12	0.7 (3)		
